# Flavonoid-derived anisotropic silver nanoparticles inhibit growth and change the expression of virulence genes in *Escherichia coli* SM10†

**DOI:** 10.1039/c7ra13480k

**Published:** 2018-01-25

**Authors:** Francis J. Osonga, Ali Akgul, Idris Yazgan, Ayfer Akgul, Renata Ontman, Victor M. Kariuki, Gaddi B. Eshun, Omowunmi A. Sadik

**Affiliations:** a Department of Chemistry, Center for Research in Advanced Sensing Technologies, Environmental Sustainability (CREATES) State University of New York at Binghamton, PO Box 6000, Binghamton, NY 13902-6000, USA; b Department of Basic Sciences, College of Veterinary Medicine Mississippi State University, P. O. Box 6100, MS 39762-6100, USA; c Department of Sustainable Bioproducts, College of Forest Resources, Mississippi State University, Box 9820, Starkville, MS 39762-9601, USA

## Abstract

We hereby present a novel greener and ecofriendly synthesis of anisotropic silver nanoparticles (AgNPs) using water soluble quercetin diphosphate (QDP). QDP was employed as a reducing, capping and stabilizing agent at room temperature without any extraneous reagents. The purpose of this study was to determine the effects of modified quercetin pentaphosphate silver nanoparticles (QPP-AgNPs) and quercetin diphosphate derived silver nanoparticles (QDP-AgNPs) on microbial growth and expressions of virulence-related genes in Escherichia coli SM10. The gene expression analysis was carried out for 12 genes which are related to virulence and stress in E. coli SM10, namely: RpoD, RpoS, ibpB, clpB, uspA, fliC, fimH, fimF, kdpE, artJ, hyaA, and gyrA. Results showed that QDP-AgNPs reduced the swarming motility by 98% which correlated with the reduction in the expression of FliC flagellar gene. A simultaneous increase in the expression of the fimbrial genes FimH and FimF that are related to motility was recorded. In contrast, treatment of the microbes with QPP-AgNPs resulted in 90% of the swarming motility at different patterns compared to QDP-AgNPs treatment for the gene expressions of motility elements. The study revealed that QDP-AgNPs up-regulated the stress related RpoD and ibpB expressions, while QPP-AgNPs up-regulated the stress related RpoS and uspA gene expressions. However, both QDP-AgNPs and QPP-AgNPs up-regulated kpdE, artJ and gry at different levels. QDP-AgNPs were also tested for their antibacterial and antifungal activities, which showed µmolar cidal activity. The growth kinetics of both Gram (—) and Gram (+) bacteria were strongly altered by QDP-AgNPs activity. Energy dispersive absorption spectroscopy (EDS) studies revealed that silver ions and/or the nanoparticles themselves transferred into bacterial cells. To the best of our knowledge, this is the first report ofstudying the genetic and kinetic response of bacteria to modified quercetin phosphate mediated silver nanoparticles and we hereby report that the molecules used to synthesize AgNPs bring about a strong effect on AgNPs manipulatory activity on the tested 12-genes

## Introduction

The combination of virulence properties and the broad antibiotic resistance profile of the E. coli strains most likely contributed to the severity of the German foodborne outbreak of 2011. ^1-3^ This outbreak could not be controlled quickly thereby resulting in over 800 cases of hemolytic-uremic syndrome (HUS) and 53 fatalities.^1-3^ The ability of pathogenic E. coli to cause infections of this type is enhanced by swarming motility that gives E. coli its ability to colonize and persist in different environments. ^3^ Swarming motility is a flagellum-dependent mechanism of translocation of bacteria on viscous substrates.^4^ Hence it is the fastest known bacterial mode of surface translocation which enables rapid colonization under nutrient-rich environ- ments.^5^ It involves the secretion of wetting agents and differentiation of bacteria into specialized, hyper-flagellated cells.^6^ Bacteria have protective or adaptive networks which modify their environments in order to survive under stressful conditions for which regulatory mechanisms, to which sigma factors,^7^ are commonly involved. Sigma factors are small proteins that bind to RNA polymerase (RNAP), and several examples of sigma factors have been shown to contribute to virulence.^8^ Two component system genes have a role in the transport of chemicals, and are identified as a regulator involved in the virulence, and intracellular survival of pathogenic bacteria. ^9^ ABC transporter complex involved in amino acid, ion or chemical transport in bacteria are essential for bacterial cellular viability, virulence and pathogenicity. ^10^

Green synthesis of silver nanoparticles (AgNPs) is an emerging research area. AgNPs have extensive properties and applications in biological applications, optical imaging, data storage, and sensing. ^14^’^15^ AgNPs exhibit good conductivity, chemical stability, catalytic, and antibacterial activity. ^16^ The large surface area of nanoparticles allows a more significant point of contact with bacteria. Furthermore, the catalytic oxidation from metallic silver and the reaction with dissolved monovalent silver ion contributes to its antibacterial activity since the metal attacks multiple targets in the organism. ^17^’^18^ Green syntheses requires three steps, the selection of appropriate solvent, selection of a non-toxic reducing agent, and selection of safe stabilizing reagent. ^19^

Typically, AgNP is prepared by the reduction of Ag^+^ using toxic chemicals such as hydrazine, sodium borohydride, hydrogen, or others. ^20^ The synthetic method we have adopted embraces a greener approach utilizing flavonoid derivatives. ^21^,^22^ We hereby report for the first time the synthesis of anisotropic AgNPs by using water soluble QDP as a reducing and capping agent using water as a solvent and at room temperature. Quercetin belongs to the class of natural products referred to as flavonoids which are capable of scavenging free superoxide radicals. They exhibit multiple biological properties including antibacterial, ^12^,^13^ cytotoxic, anti-inflammatory, and are powerful antioxidants. Quercetin is the most abundant flavonoid and has the proper structure for free scavenging activity due to the location of its hydroxyl groups. ^23-25^ However, its biological activities have been limited by poor solubility in aqueous solvents. We have reported the synthesis of phosphorylated quercetin derivatives^26^ with significantly enhanced solubility in water than the parent quercetin. ^27-29^

The anisotropic AgNPs have varying bactericidal effects when prepared in various shapes and sizes. In a wool fabric bacteria study utilizing various AgNPs, it was reported that there were far more colonies of Gram-negative E. coli on the untreated sample compared to the AgNPs treated fabrics. ^30^ Hence AgNPs supposedly eliminate bacteria by attaching to the surface of the cell membrane and disturbing the permeability and respiration functions of the cell. The silver causes the DNA to lose its replication ability and expression of ribosomal subunit proteins and other cellular proteins. The larger surface area of the smaller nanoparticles allowed for more interactions, thereby providing greater antibacterial activity. ^17^’^31^’^32^ In terms of the percent active facets of the varying nanoparticle shapes, the reactivity of silver is favored by high-atom density facets such as {111}, which is more common in the triangular nanoparticles than in the rod-like or spherical shapes. ^17^ We have studied the in vitro effects of QPP-AgNPs and QDP-AgNPs on swarming motility and proteins and enzymes in virulence of bacteria using E. coli SM10 as the model organism. QDP-AgNPs were also extensively investigated for their antimicrobial capabilities with Gram (—) and Gram (+) bacterial and fungal cells. In this work, gene expression analysis was carried out for 12 genes which are related to virulence and stress in E. coli SM10, namely: RpoD, RpoS, ibpB, clpB, uspA, fliC, fimH, fimF, kdpE, artJ, hyaA, and gyrA as shown in Table 1. This work may enable further studies on evaluation of drug-ability of flavonoid mediated AgNPs.

**Table 1:** Primers used for gene expression analysis

Gene name	**Physiological process** involved	Primer	Size	Sequence (5' → 3')	**Amplicon** (bp)	Gene ID	**Ref.**
RpoD	Stress-related genes	F	21	GATCATGAAGCTCTGCGTTG	250	947567	**In this study**
		R	21	TTCACCGATGGACATACGAC			
RpoS		F	21	GCGCGGTAGAGAAGTTTGAC	229	947210	
		R	20	GGCTTATCCAGTTGCTCTGC			
ibpB		F	21	AGCGACGATAACCACTACCG	189	948192	
		R	21	ATTTTCAGCCAGCGTAAAGC			
clpB		F	21	GGATAAAGCCATCGACCTGA	243	947077	
		R	21	CTGACGTTCTTTGTCGCTCA			
uspA		F	20	ACCGGGCTTATTGATGTGAA	191	948007	
		R	20	GACCACAAACCACCAAATCC			
flic	Flagellin	F	20	ATGGCTCCATGAAAATCCAG	234	949101	
		R	20	CTGGGTTAGTTCCGCCAGTA			
flmH	Fimbrial genes	F	20	CTTATGGCGGCGTGTTATCT	155	948847	
		R	20	CTGCTCACAGGCGTCAAATA			
flmF		F	20	TTGCTGTCACCCTGTGGTAA	174	948845	
		R	21	CGATGGAGCATTAAGGGGTA			
kdpE	Signal transduction	F	20	GCGAAGAGAGCGACAAAATC	166	945302	
		R	20	CGACGGTAACATCGGAAAAT			
artJ	ABC transporter	F	20	CCACCTATCCACCCTTTGAA	223	948981	
		R	20	GGCGTGGTAAACGATACCTG			
hyaA	Hyaluronidase enzyme	F	20	CAGGCGGAAGAAGTCTTTGA	249	945579	
		R	20	ACTTTGTCGATAGGCGTTGC			
gyrA	DNA gyrase	F	20	TGACCCGTCGTACTATTTTCG	320	946614	
		R	20	TCTTCACGGATCACTTCCATC			
GAPDH	Housekeeping gene	F	19	TCCGTGCTGCTCAGAAACG	289	947679	**Carey et al. (2008)**
		R	19	CACTTTCTTCGCACCAGCG			

## Materials and methods

Anhydrous quercetin was purchased from Indofine Chemicals Inc. (Hillsborough, NJ). Silver nitrate 99% was purchased from Sigma-Aldrich, Milwaukee, WI.

### Synthesis of QPP and QDP

QPP and QDP were synthesized and characterized as reported in our previous work. ^26^

### Synthesis of silver nanoparticles

The synthesis of isotropic AgNPs followed a procedure reported in literature^21^ with some modifications. In this work, 250 mL of 5 x 10^-3^ M AgNO_3_ was reacted with 250 mL of 5 x 10^-3^ M QPP at 40 °C. Other samples synthesized include: sample B0 was synthesized by reacting 800 µL of 4 x 10^-3^ M AgNO_3_ with 1000 µL of 5 x 10^-3^ M QDP while B1 was synthesized by reacting 1000 µL of 4 x 10^-3^ M AgNO_3_ with 1000 µL of 5 x 10^-3^ M QDP at 40 °C. Sample S1 was synthesized by reacting 500 µL of 5 x 10^-3^ M AgNO_3_ with 300 µL of 5 x 10^-3^ M QDP. S5 was prepared by reacting 500 µL of 5 x 10^-3^ M AgNO_3_ with 500 µL of 5 x 10^-3^ M QDP. S2 to S4 were synthesized by reacting QDP with AgNO3 in the ratio of 1: 2, 1: 3, 2: 3 for S2, S3 and S4 respectively. S5 was prepared by reacting 500 µL of 5 x 10^-3^ M AgNO_3_ with 500 µL of 5 x 10^-3^ M QDP. Sample A was synthesized by reacting 800 µL of 4. 5 x 10^-3^ M AgNO_3_ with 800 µL of 5 x 10^-3^ M QDP at room temperature while B and C were synthesized by reacting 5 x 10^-3^ M AgNO_3_ with 5 x 10^-3^ M QDP in a ratio of 1: 2 and 1: 3 respectively. All the reactions were carried out at room temperature and monitored by both UV-Vis and color changes. The colloidal AgNPs formed were sonicated for 25 minutes in an ultrasound bath and were then centrifuged 3500 rpm for 20 minutes to obtain pellets. The pellets formed were washed three times using Nanopure® water and were used for characterization.

### Characterization

UV/Vis absorption spectra were carried out on a HP 8453 UV-visible diode array spectrophotometer. Transmission electron microscopy (TEM) measurements were carried out on a JEOL TEM 2100F.

### Bacterial growth

E. coli strain SM10 was grown at 37 °C using Luria-Bertani (LB) broth and agar (Difco). Ampicillin (100 mg mL^-1^) was added to the growth media to sustain bacterial strain. All the strains were maintained at -80 °C in LB broth with 20% glycerol (Sigma-Aldrich, Mexico). Prior to use, an aliquot of frozen culture was used to inoculate LB agar tubes, incubated for 48 h at 37 °C then stored at 4 ^°^C. For assays, an aliquot of growth media was transferred to 5 mL Luria-Bertani broth tubes (LB, EMD Milli-pore Corporation, Germany) and incubated for 24 h at 37 ^°^C. In this study, toxicity of QDP silver nanoparticles (S1 to S3 were coded as QDP-AgNP1 to QDP-AgNP3 while samples S5 and C were coded as QDPAgNP4 and QDPAgNP5 respectively) were tested on Escherichia coli (E. coli) 25922, Enterobacter aerogenes (E. coli ATCC® 87423), Staphylococcus epidermidis 12228 (S. epidermidis), Citrobacter freundii 8090 (C. freundii) bacteria, and Trichaptum biforme (T. biforme) and Aspergillus nidulans (A. nidulans) fungi. For gene expression studies, 2. 5 mM of compounds were used in this experiment. The bacteria growth in 2 mL LB broth, and 5 µL of E. coli SM10 was added on the culture. 3 tubes of non-treated cultures were used as a control for each bacterial group.

### Preparation of samples for SEM

10 µM of QDP-AgNPs were used for 10^4^ cfu mL^-1^ bacterial and 10^4^ cells per mL inoculums. 24 h incubation was performed to provide enough time for nanoparticle-cell interactions. This was followed by 20 µL from the sample tubes which were dropped on top of the 0. 2 µm pore-size Whatman® and then left to dry for 3 days at room temperature. Samples were then coated with 3 nm gold-layer.

### Swarming motility test

Swarming motility was carried out by following the procedure described in the literature. ^11^ Briefly, soft LB agar (0. 35% agar) plates containing various concentrations of 2. 5 µM of compounds were prepared 24 h prior to use. For the assay, 5 mL aliquot of cultures (1 x 10^8^ cfu mL^-1^) was inoculated in the center of the Petri dish and incubated for 24 h at 37 ^°^C. The degree of swarming was based on the migration distance from the center measured in cm.

### Total RNA isolation and cDNA preparation

A single E. coli colony was inoculated in 5 mL of LB broth, followed by 16-18 h incubation at 37 °C with shaking. An aliquot (400 mL) was inoculated into fresh 4 mL tubes of LB broth including the tested compounds and incubated for 2 h at 37 ^°^C with shaking (120 rpm, MaxQ Mini 4450, Barnstead/Lab-Line), and then was followed by immediate extraction of RNA.

Total RNA was isolated from four biological replicates by using RNeasy Mini Kit (Qiagen). Contaminating bacterial DNA was eliminated by DNase I treatment with RNase-Free DNase Set (Qiagen). The quality and concentration of the isolated total RNA were measured by Nano Drop 1000 (Thermo Scientific). First-strand cDNA was produced from 1 mg total RNA using Maxima First Strand cDNA Synthesis Kit for RT-qPCR (Thermo Scientific).

### Real-time qPCR

For gene-expression analysis, quantitative real-time PCR (qRT-PCR) was performed using a Mx3005P qPCR System (Agilent Technologies, CA, USA) and DyNAmo SYBR Green qPCR Kit was used (Finnzymes Oy, Espoo, Finland). Each 20 µL PCR reaction contained 10 µL SYBR Green 2x mix, 0. 2 µM each of forward and reverse primers (Table 1), and 1 µL of 100x diluted cDNA. The PCR was set to initial denaturation at 95 ^°^C for 3 min, 45 cycles of denaturation at 95 °C for 15 s, annealing at 60 °C for 30 s, and extension at 72 °C for 30 s, and a final extension at 72 ^°^C for 3 min. At the end of the PCR, a melting curve program from 60 °C to 95 °C with 0. 5 °C increase every 15 s were run. The assessment was carried out by PCR to determine the suitability of primers and experimental conditions. We used glyceraldehyde-3-phosphate dehydrogenase (GAPDH), a housekeeping gene as an internal control. A sample from untreated condition was set as a calibrator in each experiment. Relative expression rates were calculated by the threshold cycle changes in sample and calibrator. The 2^—ΔΔCt^ method^33^ was used to calculate the expression level of related genes. All expression values were normalized against GAPDH. ^ΔΔC^^t^ was calculated by AAC_t_ = ΔC_t_ (compound added condition) — ΔC_t_ (non-treated condition), where ΔCt is the normalized signal level in a sample (ΔC_t_ = C_t_ of target gene — C_t_ of reference gene). The one-way Analysis of Variance (ANOVA) test was used to compare gene expression among conditions. P value selected for statistical significance was <0. 05.

## Results and discussion

### Optical and microscopic characterization of synthesized silver nanoparticles

**Fig. 1:**
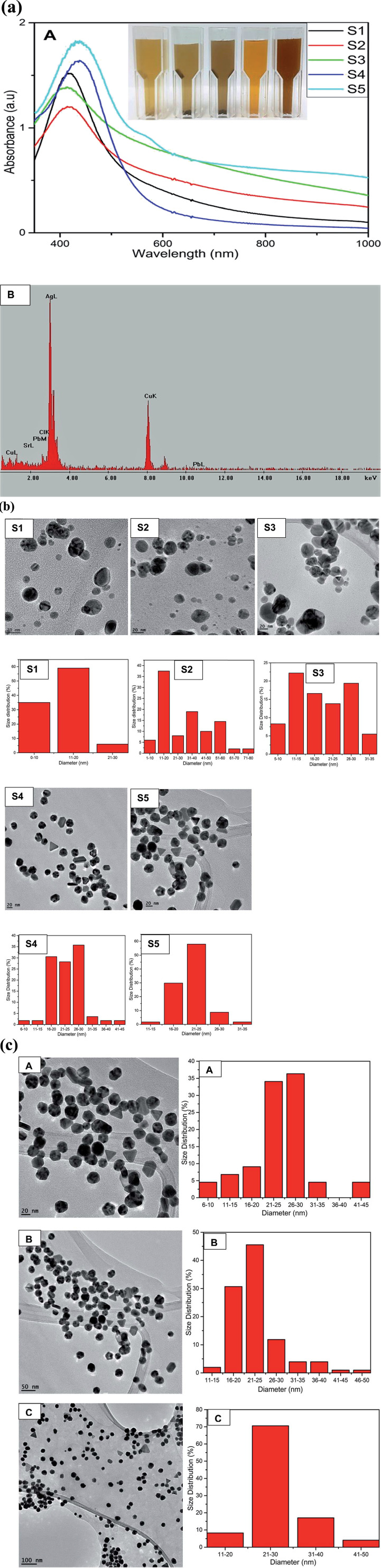
(a) (A) UV-Vis spectra of silver nanoparticles at different concentrations of QDP (S1-S5). Inset pictorial images of AgNPs after S1 (B) EDS of AgNPs obtained from sample S4 confirming the formation of elemental Ag. (b) TEM images of AgNPs for samples S1-S5 and the corresponding histograms showing size distribution. (c) TEM images at different magnification (A) 20 nm, (B) 50 nm and (C) 100 nm scale bar of AgNPs for samples S5 and the corresponding histograms showing size distribution.

The formation of AgNPs was monitored and confirmed by using UV-Vis spectroscopy to assess the signature surface plasmon resonance (SPR) bands. Fig. 1a shows the UV-Vis spectra of the formation of AgNPs with one SPR band for samples S1 to S4 ranging from 438 nm to 453 nm clearly demonstrating the formation of AgNPs and indicating the isotropic nature of the nanoparticles.

Transmission electron microscopy (TEM) demonstrated the spherical nature with average sizes of 11. 89 nm, 19. 23 nm, 30. 60 nm and 23. 93 nm for S1, S2, S3 and S4 respectively. Sample S5 with an average size distribution of 25. 68 nm exhibited two SPR peaks at 446 nm and 584 nm due to transverse and longitudinal SPR modes. ^34^ The TEM images of S5 clearly exhibited the anisotropic nature of AgNPs by the formation of nanoprisms (Fig. 1b). The color of AgNPs for samples S1 to S5 show the formation of AgNPs at different reaction ratios of QDP and AgNO_3_ and hence gave different SPR peaks which are in agreement with the size analysis of TEM images as shown by the histograms in Fig. 1b. Fig. S1† show the formation of SPR bands at 434 and 431 nm for B0 and B1 respectively. The formation of reddish brown color signaled the formation of AgNPs with the morphology revealed to be spherical (Fig. S1†). The energy dispersive absorption spectroscopy (EDS) confirmed the formation of elemental Ag as seen with the formation of strong Ag signal at 3 keV in Fig. 1a-B. The presence of copper could have originated from the TEM grid. The increase in the concentration of the precursor led to increase in size of the spherical AgNPs for samples S1 to S3 (Fig. 1b). However, in sample S4 (56 nanoparticles) at a ratio of 2: 3 for QDP to AgNO_3_ the size decreased and spherical nature drastically changed and small triangles commenced to form as shown in Fig. 1b. In sample S5 that was prepared by reacting 500 µL of 5 x 10^—3^ M AgNO_3_ with 500 µL of 5 ⨯ 10^—3^ M QDP at room temperature for 2 hours, silver nanoprisms were formed as depicted by TEM S5 (Fig. 1b) in which 82 nanoparticles were counted.

The anisotropic nature of sample S5 is further demonstrated by the TEM images taken at different magnifications of sample S5 and the corresponding size distribution in which the number of nanoparticles were 88, 101 and 170 nanoparticles with average size of 24. 80 nm, 25. 29 nm and 27. 60 nm for A (20 nm scale bar), B (50 nm scale bar) and C (100 nm scale bar) respectively as shown in Fig. 1c. The formation of triangles, truncated triangles and hexagonal is clearly depicted by TEM image in Fig. 1b(S5), c and 2 as reported by other previous anisotropic synthetic methods. ^20^ The small spherical AgNPs can still be seen confirming that the growth of the nanoprisms began by the formation of spherical nanoparticles which acted as the nuclei for the growth to anisotropic form. It is worth noting that the SPR peaks remained unchanged even after 8 months indicating the AgNPs were very stable. The main aim of this synthetic work was to develop anisotropic AgNPs at room temperature. Therefore we refined the conditions for the synthesis as described in the procedure section.

**Fig. 2:**
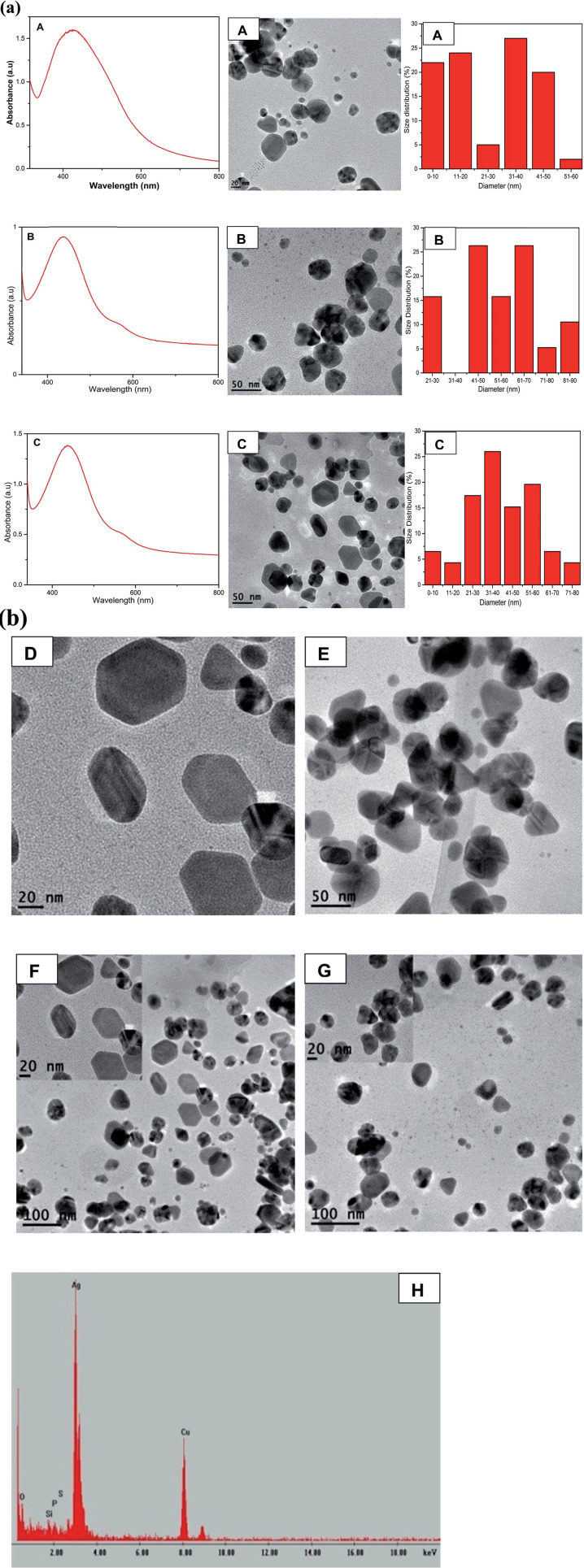
(a) UV-Vis spectra of the anisotropic AgNPs (A to C) with their TEM images A, B, C and corresponding histograms illustrating size distribution. (b) High resolution TEM images D and E for sample C and B respectively. F and G are TEM images of sample B and C respectively while inset in F and G are HRTEM images of the AgNPs. EDS (H) for AgNPs of sample C.

Fig. 2a shows the formation of anisotropic AgNPs with two SPR bands at 438 nm and 584 nm. As the concentration of QDP increased, the average sizes of AgNPs were found to be 25. 80 nm, 55. 96 nm, 39. 67 nm for samples A, B, and C respectively (Fig. 2a). This could be attributed to the strong reducing and stabilizing power of the flavonoid derivatives. ^21^,^22^ TEM images for B (AgNO3: QDP at a ratio of 1: 2) and C (AgNO3-: QDP at a ratio of 1: 3) show the formation of Ag nanoprisms. The increase in the concentration of QDP led to the formation of anisotropic AgNPs with well-refined edges as shown in Fig. 2a-C and b-D-G. EDS spectra confirmed the presence of silver as shown in Fig. 2b-H. This was due to the fact that Ag+ was reduced to Ag^0^ by the QDP macromolecules.

### Effect on swarming motility

**Fig. 3:**
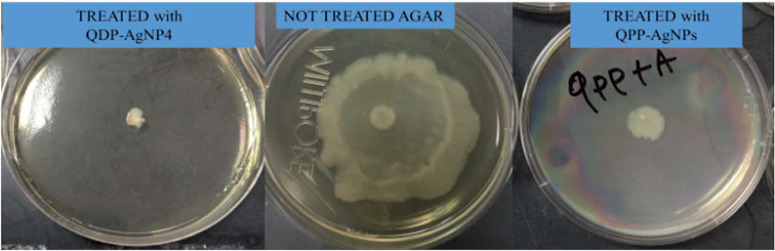
The reduction (66%) on the swarming motility of E. coli by the effect of QDP-AgNP4 while no significant difference was determined in the QPP-AgNPs treated group. A negative control group served as the baseline.

A very distinct swarming motility was observed for QDP-AgNPs treated conditions. It was observed that QDP-AgNPs reduced the swarming motility at over 60% with 2. 5 µM concentration (Fig. 3). On the other hand, in QPP-AgNPs exposed agar, there was no significant difference compared to control soft agar motility assay.

### Gene expression

**Fig. 4:**
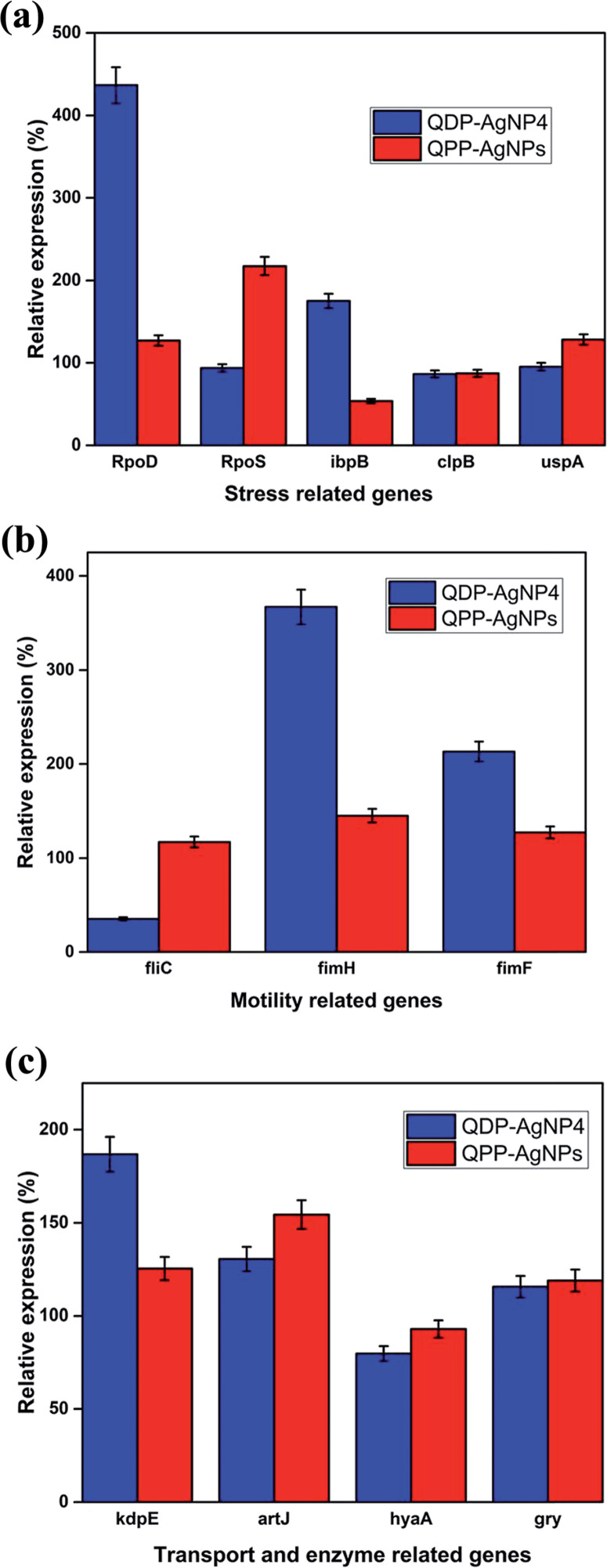
(A) The gene expression of stress-related genes for E. coli strains after the addition of QPP-AgNPs and QDP-AgNP4. (B) The gene expression of motility-related genes for E. coli strains after the addition of QPP-AgNPs and QDP-AgNP4. (C) The gene expression of virulence-related genes for E. coli strains after the addition of QPP-AgNPs and QDP-AgNP4.

AgNPs has been reported for their gene-expression manipulating capabilities. Fig. 4A depicted the influence of QDP-AgNP4 and QPP-AgNPs on the expression of five stress related genes RpoD, RpoS, IbpB, ClpB, and UspA in E. coli. The expression of the genes showed an increase in the expression of the RpoD and IbpB in the QDP-AgNP4 treated group. Bacteria create a protective or adaptive network to assist microorganisms to modify their environments and/or to survive the stress condition, and a common regulatory mechanism involves sigma factors. ^7^

Sigma factors are small proteins that bind to RNA polymerase (RNAP). We have tested the gene expression of sigma factors RpoD and RpoS. The study revealed that RpoD was upregulated in response to QDP-AgNP4 treatment, while RpoS was upregulated in QPP-AgNPs in comparison to the control group and the QDP-AgNP4 treated group (Fig. 4A). The rpoS gene is involved in the regulation of more than 50 genes in Gram-negative bacteria, ^11^ so its expression and regulation is complex.

Furthermore, both swarming formation are also under complex genetic regulation. Hence it may be extremely difficult to establish any clear relation between rpoS expression and these phenotypic characteristics. RpoD has housekeeping function, and is used for the detection of bacterial viability and development in the infected tissue to study host-pathogen interactions. ^36^

In our study, using the QDP-AgNP4 group, a significant increase in gene expression was determined. Flagella are central in the bacterial virulence of E. coli that provide motility and contribution to colonization of host cells, and penetration of the mucosal layer. ^37^ Flagella expression is often coregulated with the expression of other virulence factors. ^37-40^ Whereas a significant decrease was observed for the expression of fliC, a flagellar gene; fimbria genes fimH and fimF were upregulated in QDP-AgNP4 treatment (Fig. 4B).

There was no significant difference determined in QPP-AgNPs treatment in contrast to over 90% decrease in swarming motility. In the case of QDP-AgNP4 treatment, the expression of fliC was significantly down regulated, which correlated with swarming assay. Furthermore, an increase in virulence gene expression which included fimH, fimF, kdpE, and art] was determined.

KdpD/KdpE is a two component system gene that has a role in potassium (K**^+^**) transport and identified as a regulator involved in the virulence and intracellular survival of pathogenic bacteria. ^9^ It also plays a key role in turgor pressure and salt shock protection. Artj is a part of the ABC transporter complex which is involved in arginine transport by binding l- arginine with high affinity. Hereby it was found that the two transporter genes were affected by the tested nanoparticles, and they were both upregulated (kdpE and art]) (Fig. 4C) in QDP-AgNP4 and QPP-AgNPs treated group when compared to controls.

The results obtained from this study are very promising and future research will be based on comparison with commercial drugs in order to investigate whether flavonoid derived AgNPs could be used as alternative to antibiotic-based approaches to control pathogenic bacteria. This could have significant potential in the treatment of acquired antibiotic resistance that is posing major public health concern.

### Antimicrobial effect of AgNPs

**Fig. 5:**
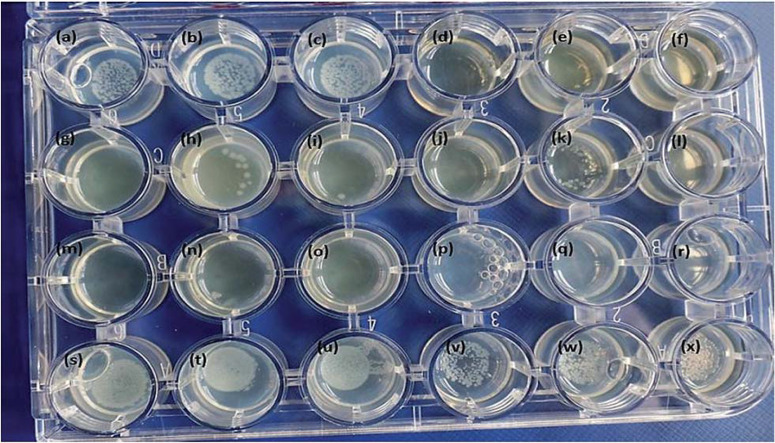
All nanoparticles were tested at 10 ^5^ M, which were dropped on top of the inoculum in 25 µL. (a) QDP-AgNP1, (b) QDP-AgNP2, (c) QDP-AgNP3, (d) QDP-AgNP4, (e and f) QDP-AgNP5 for 1000 cfu in 20 µL; (g) QDP-AgNP1, (h) QDP-AgNP2, (i) QDP-AgNP3, (j) QDP-AgNP4, (k and l) QDP-AgNP5 for 100 cfu in 20 µL; (m) QDP-AgNP1, (n) QDP- AgNP2; (o) QDP-AgNP3; (p) QDP-AgNP4; (q and r) QDP-AgNP5 for 50 cfu in 20 µL; (s) QDP-AgNP1, (t) QDP-AgNP2, (u) QDP-AgNP3, (v) QDP-AgNP4, (w and x) QDP-AgNP5 for 10 000 cfu in 20 µL.

The anisotropic silver nanoparticles analyzed in this work showed significant bactericidal activity against all strains tested. Fig. 5 clearly demonstrates that QDP-AgNP2 had the lowest antibacterial activity since it only showed its antibacterial activity against 50 cfu E. coli. Fig. 5k clearly demonstrated that there was no bacterial growth in the center and this could be due to the fact that QDP-AgNP4 did not spread to the edges.

This is because at higher concentrations, no bacterial growth was observed for QDP-AgNP4 treatment.

Even though, QDP-AgNP-4/-5 showed very strong antibacterial activity towards E. coli, in the case of 10^4^ cfu E. coli in 20 µL inoculum, only QDP-AgNP4 showed over 99% elimination. Because the numbers of colonies formed were lower than the inoculum, it can be speculated that QDP-AgNPs showed bactericidal activities. Similar trends were observed for QPP- AgNPs for their bacteriostatic and bactericidal activities. ^21^ Furthermore, medicinal plants^12, 13^ and their derived AgNPs studies have reported strong antibacterial activity against E. coli. ^41-43^

QDP-AgNP4 (Fig. S2†) and QDP-AgNP5 (Fig. S3†) were further tested in liquid cultures. QDP-AgNP4 was tested at 10 and 100 µM for E. coli, S. epidermidis and C. freundii, where 100 µM treatments were observed for 100% bactericidal activity. QDP- AgNP5 was tested from 5 to 100 µM concentrations for E. coli 25922 and E. coli 87423; at 50 µM and 100 µM concentrations and total bactericidal activity was observed. At 5, 20 and 30 µM levels, E. coli 25922 showed relatively higher viability in comparison to E. coli 87423. Fig. S4† reveals that QDP-AgNP4 and QDP-AgNP5 showed similar toxicity on E. coli 25922 and E. coli 87423 for their 10^3^ cfu mL^—1^. Comparison of Fig. S3 and S4† revealed 10-times decrease of bacterial inoculum. Thus strongly enhancing the observed antibacterial activity for same QDP-AgNP5 concentrations (i.e. 5 µM), which correlated with the observations seen in Fig. 5. It is imperative to note that at the tested bacterial concentrations (i. e. 10^3^ cfu mL^—^^1^), 1 µM Ag+ ion did not cause the expected bactericidal activity which might be related to the formation of AgNPs from the Ag^+^ ions in the media (results not shown). Even though at initial incubation periods, Ag^+^ ions showed stronger antibacterial activity in comparison to QDP-AgNP4 and QDP-AgNP5 treatment, it was found that with time formation of nanoparticles decreased the observed overall antibacterial activity. However, in contrast, QDP-AgNP4 and QDP-AgNP5 showed reverse antibacterial activity.

**Fig. 6:**
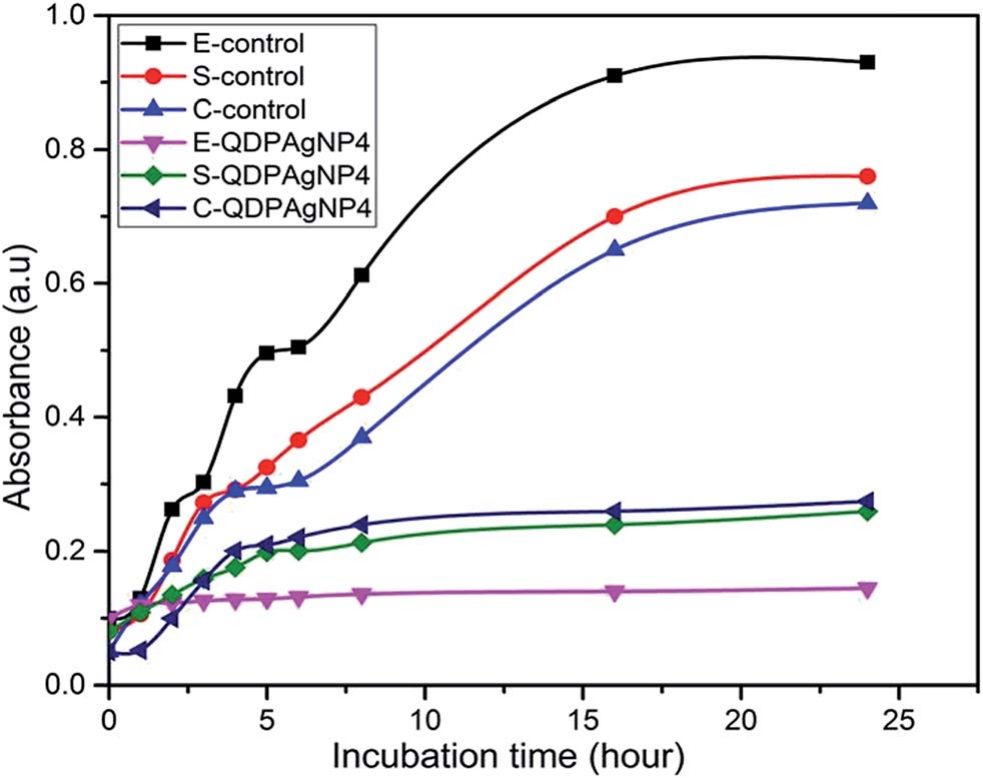
E refers to E. coli; S refers to S. epidermidis; C refers to C. freundii. QDP-AgNP4 had strong impact on growth kinetics of the three bacteria. 1 µmM of QDP-AgNP4 was used in all cases, where the initial bacteria concentrations were 10^4^ cfu mL^-1^.

In the cases of E. coli and S. epidermidis, nearly no growth was observed (Fig. 6) in comparison to control, any characteristic region related to growth pattern was not observed. Since there was growth for both bacteria, it can be speculated that QDP-AgNP4 extended the lag-phase. In actual fact, no significant growth of E. coli and S. epidermidis were observed even at 1 week incubation. This implies that QDP-NP4 drove E. coli and S. epidermidis into infinite lag-phase. However, for C. freundii, a short log-phase and extended stationary phase were observed, which might be related to the time-dependent toxicity of QDP-AgNP4. Similarly, Raghupathi et al. (2011) reported that zinc nanoparticles extended the lag-phase and reduced log-phase of S. aureus. ^35^

QDP-AgNP4 (Fig. S5-S7†) and QDP-AgNP5 (not shown) were used to evaluate antibacterial activity of the AgNPs. Based on SEM and EDS results, it can be speculated that AgNPs and/or the Ag^+^ formed from the nanoparticles posed their toxicity with or without being transferred into the inner-cellular area, which was observed to be independent of Gram properties of the tested bacteria.

**Fig. 7:**
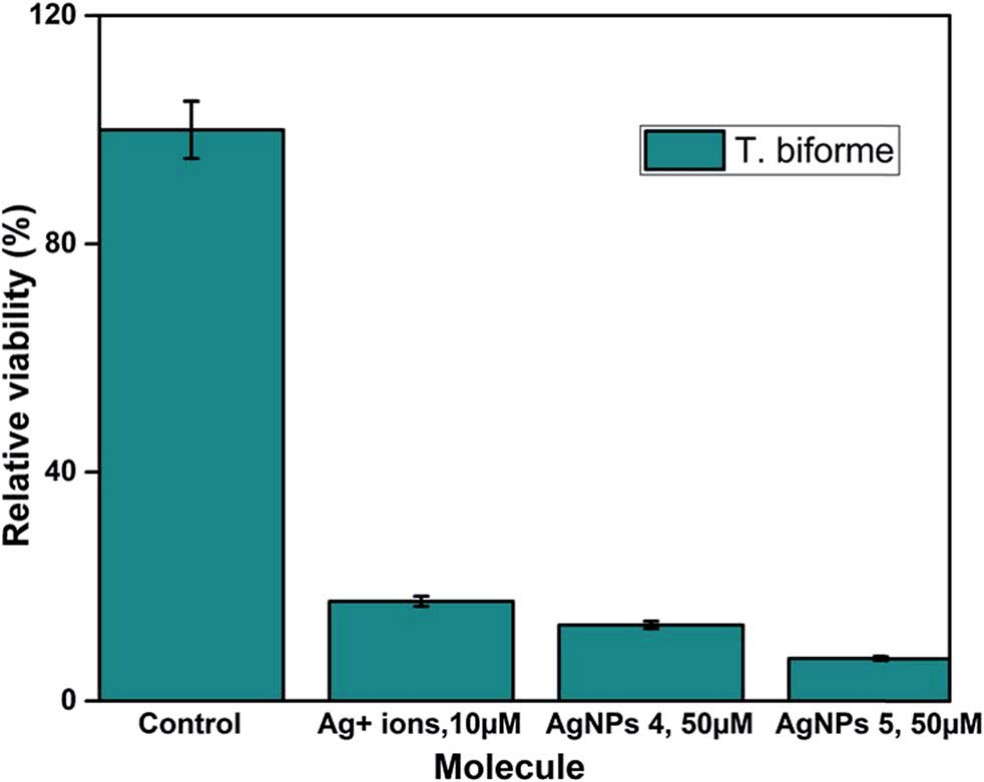
10^4^ cells per mL of Trichaptum biforme was grown in YPD- broth. Silver ions were used 1/5 of nanoparticle concentrations.

Fig. S8† shows that QDP-AgNP4 totally eliminated T. biforme growth at 100 µM treatment. Cytotoxicity of QDP-AgNP4 was observed relatively stronger for Aspergillus nidulans. The goal was to determine the overall comparison between QDP-AgNPs and silver ions. The results revealed that T. biforme formed AgNPs within 3 days from the Ag+ ions in media and it can be speculated that Trichaptum biforme drove Ag^+^ to form AgNPs, which can be a way of its defense mechanism against possible silver poisoning. It was observed that QDP-AgNP4 did not eliminate the entire Trichaptum biforme, which was shown before for the same concentration of QDP-AgNP4. At Fig. S8, † QDP-AgNP4 was 1 week old while for the results shown in Fig. 7, it was 2 months old. Similar results were obtained for the antibacterial test, where the same amount of one-week old QDP- AgNP4 showed total elimination, two months old QDP-AgNP4 required up to 10 times amount to eliminate the same amount of bacteria. Incubating AgNPs at room temperatures over several months generally, leads to decrease in antibacterial activity which might be related to agglomeration. ^41^

QDP-AgNP-1/-5 were tested on Gram (—) and Gram (+) bacteria and two fungi species. The findings revealed that QDP- AgNP4 showed the best antimicrobial properties when it is freshly prepared while its antimicrobial activity decreased by up to 10 times when the nanoparticles were left at room temperature for 2 months.

Although QDP-AgNPs and QPP-AgNPs^21^ showed similar antibacterial capabilities, their effect on genetic expression for the tested genes obtained was very distinct. Both RpoD and RpoS mediate the RNA polymerase binding to the same promoters; ^45^ while QDP-AgNPs up-regulated RpoD expression over 4 times, it down-regulated the RpoS expression 7%. In contrast, QPP-AgNPs up-regulated the expression of RpoD and RpoS as 27% and 117%, respectively. Expressions of ibpB, a protective heat shock protein against stressors include oxidative stress, ^46^ up-regulated as 75% by QDP-AgNPs and down-regulated as ˜50% by QPP-AgNPs. Over-expression of IbpB with Ag(I) treatment was previously reported, ^47^ but here down-regulation of IbpB is unique which is a sign of that QPP was either driving the activity of QPP-AgNPs or freed-QPP directly acting on the gene modulation. Previously we showed that E. coli can boost up its metabolism to fight against AgNPs mediated stress. ^44^ In contrast to this, QDP-AgNPs down- regulated the FliC gene by 65% which was tried to be compensated by over-expression of FimH (3. 6 times) and FimF (2. 1 times) while QPP-AgNPs up-regulated expression of all the three by 17%, 45%, and 27%, respectively. However, the expression of the tested transport enzyme showed the similar trend in response to both QDP-AgNPs and QPP-AgNPs treatments. The difference in the shapes^48, 49^ between QDP-AgNPs (anisotropic as shown in sample S5 Fig. 1b and c) and QPP (spherical as shown in Fig. S1†) which might be the reason behind the alterations in the expressions of the tested genes.

## Conclusion

We have successfully synthesized anisotropic AgNPs of unique shapes using modified quercetin phosphate derivatives at room temperature without using toxic reducing and capping agents. In this work QDP and QPP acted as reducing agent, capping and stabilizing agent. The exposure to different concentrations of QDP-AgNP4 and QPP-AgNPs resulted in variations in the phenotypic traits and expression of virulence factors. QDP-AgNPs and QPP-AgNPs did not show a recognizable difference, but the shapes were different which might be the reason behind the alterations in the expressions of the tested genes. Besides, the kinetics of Ag^+^ releases from QDP-AgNPs and QPP- AgNPs might be another reason contributing to the alterations. Apart from the nanoparticles and Ag^+^ releases kinetics, the unleashed QDP and QPP might also have taken place during the alteration. Based on the genetic studies and comparative antimicrobial studies, it can be concluded that QDP-AgNPs gave dramatic effect on in vitro pathogenicity of E. coli SM10 in addition to its strong antimicrobial capability including antibacterial and antifungal. Furthermore, antibacterial tests of QDP-AgNP4 revealed that the growth kinetic of both Gram (—) and Gram (+) bacteria were strongly altered by extending lag phase and/or reducing the log phase. Besides, QDP-AgNPs were compared with free Ag^+^ ions for their toxicity on bacteria and fungi, where long-term Ag^+^ ions toxicity was observed to diminish because of AgNPs formation. The findings can open a new window to study and understand the possible antimicrobial mechanisms of flavonoid mediated-synthesized nanoparticles in drug design.

## Conflicts of interest

There are no conflicts to declare.
