# Supplementary material for: Flavonoid-derived anisotropic silver nanoparticles inhibit growth and change the expression of virulence genes in Escherichia coli SM10
Source: RSC Adv. 2018 Jan 25;8(9):4649–61. doi: 10.1039/c7ra13480k (PMC7745121; doi:10.1039/c7ra13480k)
Supplement: Supplementary file 1 [file RSCA-08-09-4649-s001.pdf]

Electronic Supplementary Material (ESI) for RSC Advances.
This journal is © The Royal Society of Chemistry 2018

SUPPORTING INFORMATION

Flavonoid-derived anisotropic silver nanoparticles inhibit growth and change the expression of virulence genes in *Escherichia coli* SM10

Francis J. Osonga, [a] *Ali Akgul ,* [b] *Idris Yazgan,* [a] *Ayfer Akgul,* [c] *Renata Ontman,* [a] *Victor M. Kariuki,* [a] *Gaddi B. Eshun* [a] *& Omowunmi A. Sadik* [a*]

[a] Department of Chemistry

Center for Research in Advanced Sensing Technologies & Environmental Sustainability (CREATES)

State University of New York at Binghamton

P.O Box 6000 Binghamton, NY, 13902.

[b] Department of Sustainable Bioproducts, College of Forest Resources, Mississippi State University, Starkville, MS, USA.

c. Department of Basic Sciences, College of Veterinary Medicine Mississippi State University, Starkville, MS, USA

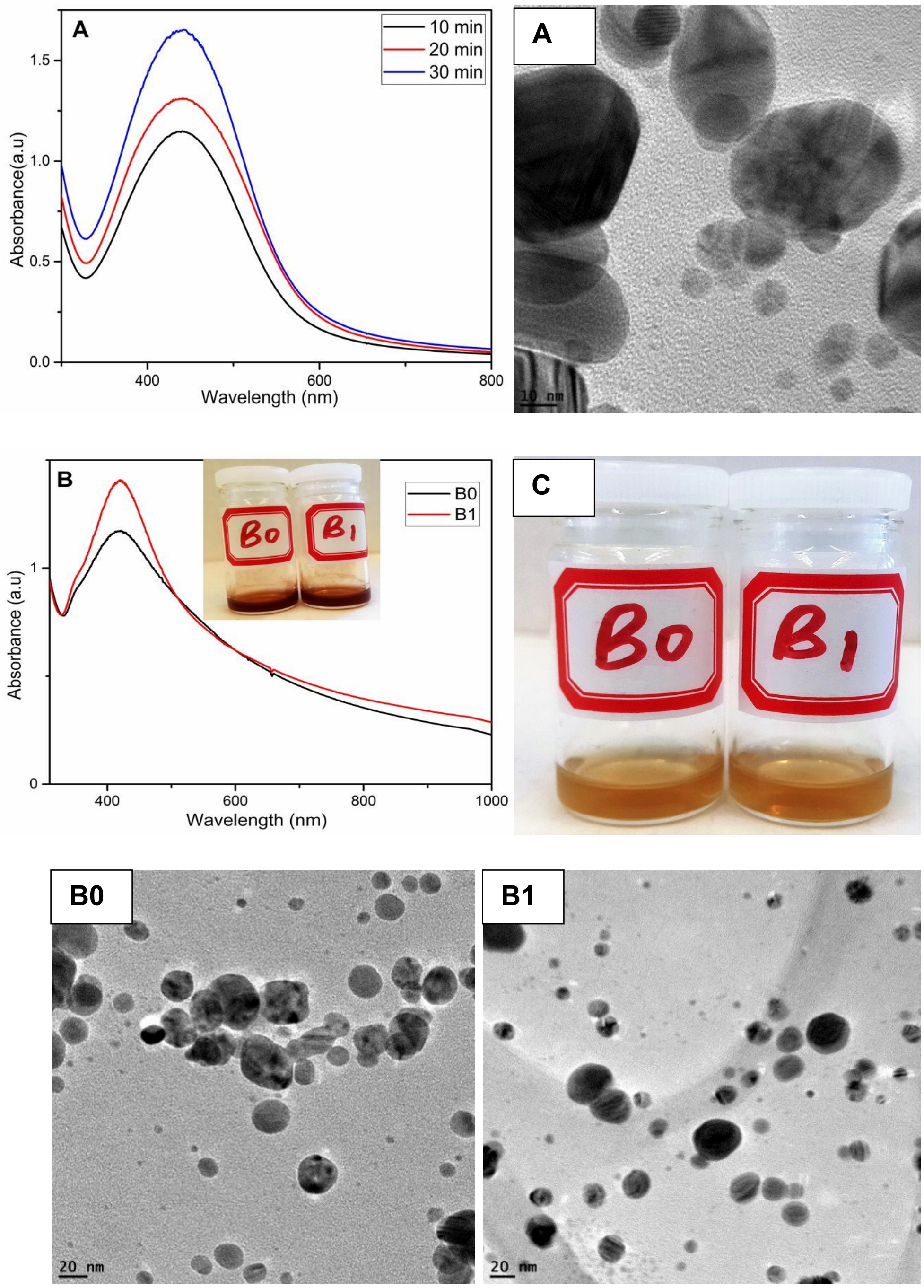

Figure S1: Uv-Vis characterization spectra of AgNPs mediated by reacting 250μL of $5x10^{-3}$ M $AgNO_3$ with 250μL of $5x10^{-3}$ M QPP at 40°C (A) indicating reaction as time progressed. Sample B0 was synthesized by reacting 800μL of $4x10^{-3}$ M $AgNO_3$ with 1000μL of $5x10^{-3}$ M QDP while B1 was synthesized by reacting 1000μL of $4x10^{-3}$ M $AgNO_3$ with1000μL of $5x10^{-3}$ M QDP heated to 40°C and reaction allowed to proceed for 25 minutes. Inset shows the formation of reddish brown color for Bo and B1 respectively while C shows the color of sample Bo and B1 immediately after mixing. TEM images (A) for QPP derived AgNPs formed in A while Bo and B1 for QDP derived AgNPs B0 and B1 respectively.

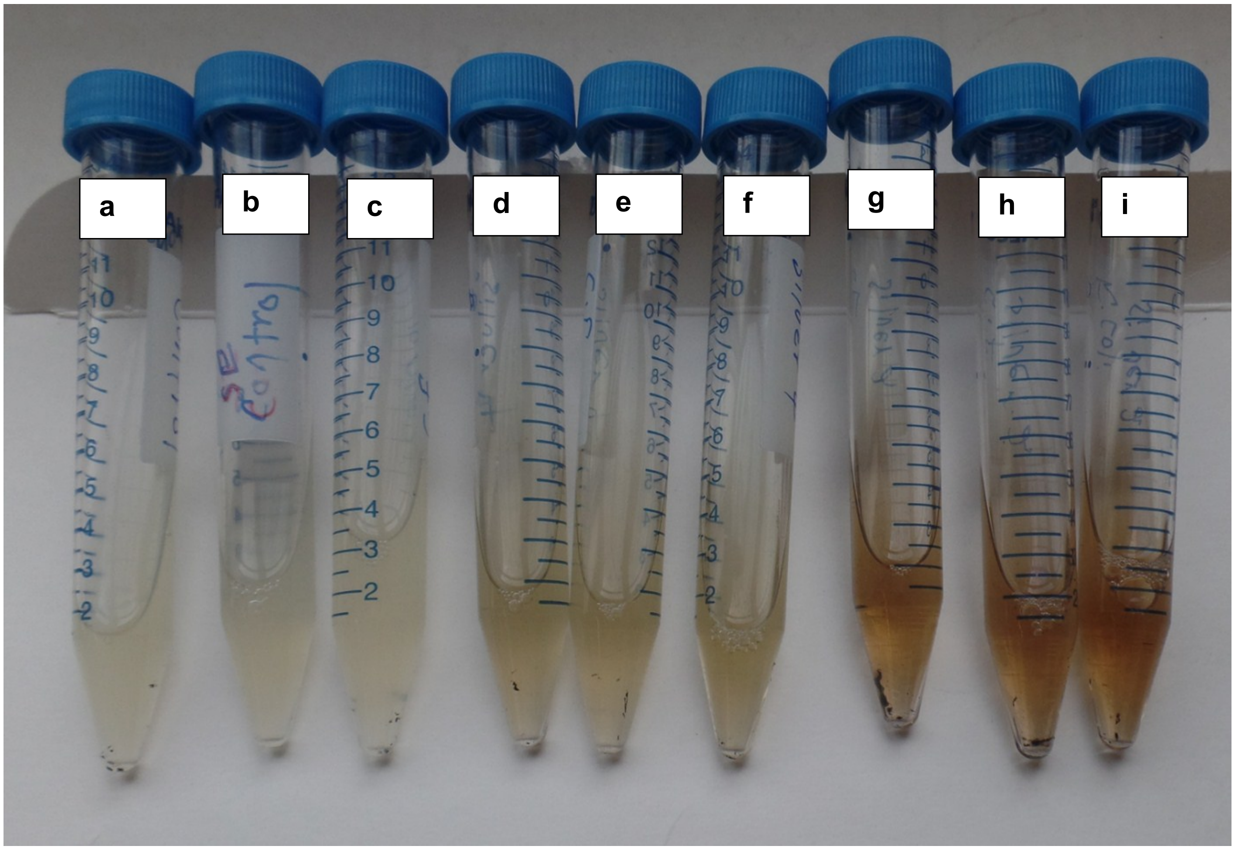

Figure S2: Controls of (a) *E.coli*, (b) *S.epidermidis* and (c) *C.freundii* while 10^{-5}M and 10^{-4}M of QDP-AgNP4 in (d and g) *E.coli*, (e and h) *S.epidermidis* and (f and j) *C.freundii*, respectively. In all cases, bacterial concentrations were 10^4 cfu/mL; while 10^{-4} M concentration of QDP-AgNP4 eliminated the growth of bacteria at 10^{-5} M suppression of growth was below 90%. As expected, in liquid cultures, survival of bacteria against nanoparticles is higher than in solid cultures. When 20 μL inoculum was taken from the tubes treated with 10^{-4} M and incubated on plain Nutrient Agar, 750 cfu/mL growth was observed within two weeks. These observations confirmed the results obtained for **Figure 5**, indicating that the silver nanoparticles possessed bactericidal activity.

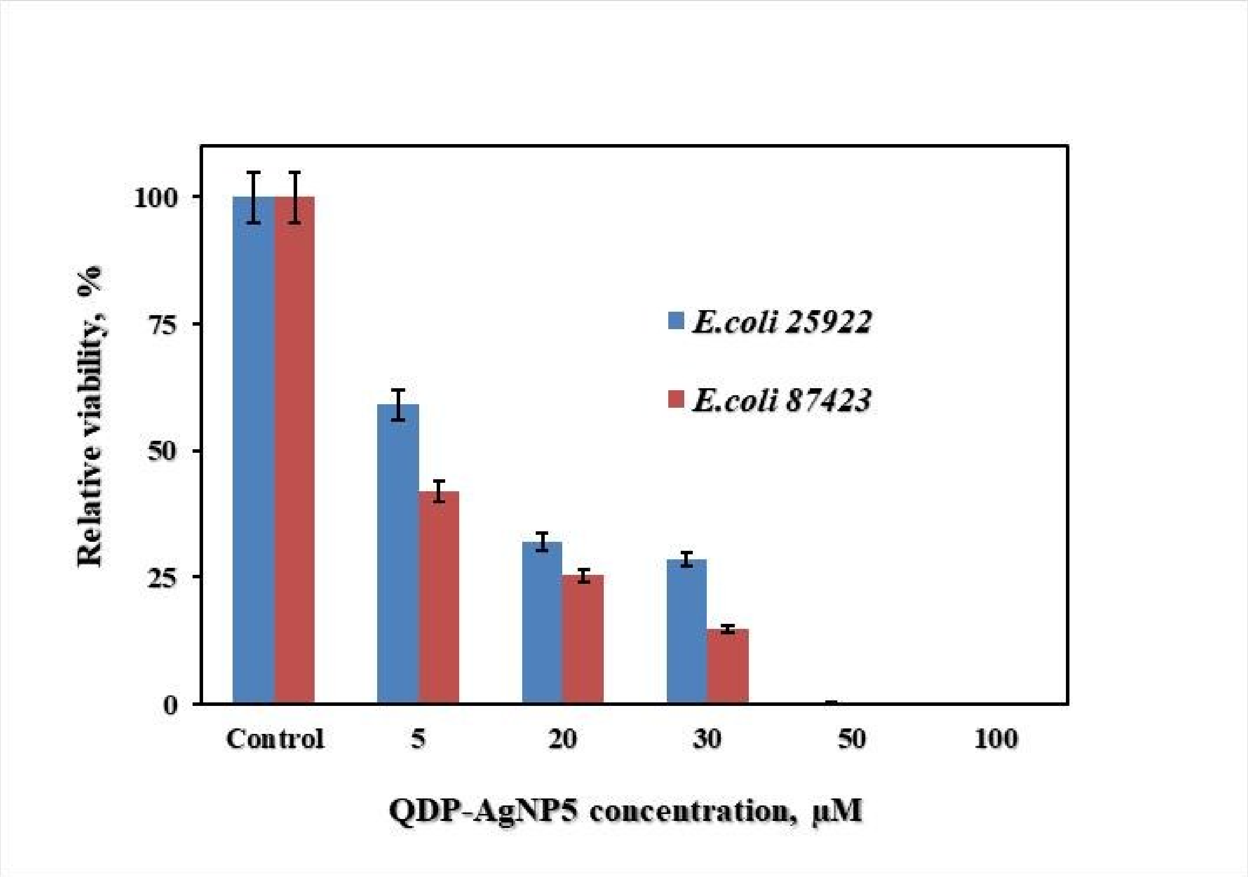

Figure S3: QDP-AgNP5 nanoparticles were tested in liquid cultures at 10^4 cfu/mL *E.coli* 25922 and *E.coli* 87423. In contrast to *E.coli* 25922, the nanoparticles showed greater effects on *E.coli* 87423. At 50 and 100 µM concentrations, no bacterial growth was observed. The turbidity at 600 nm for blank and the tubes treated with 50 and 100 µM QDP-AgNP5 were the same. 20 µL from the tubes was inoculated onto Nutrient Agar to test the bactericidal and bacteriostatic activities at different concentrations. Two-week incubation showed that no bacterium survived, at least to cause colony formation under the test conditions.

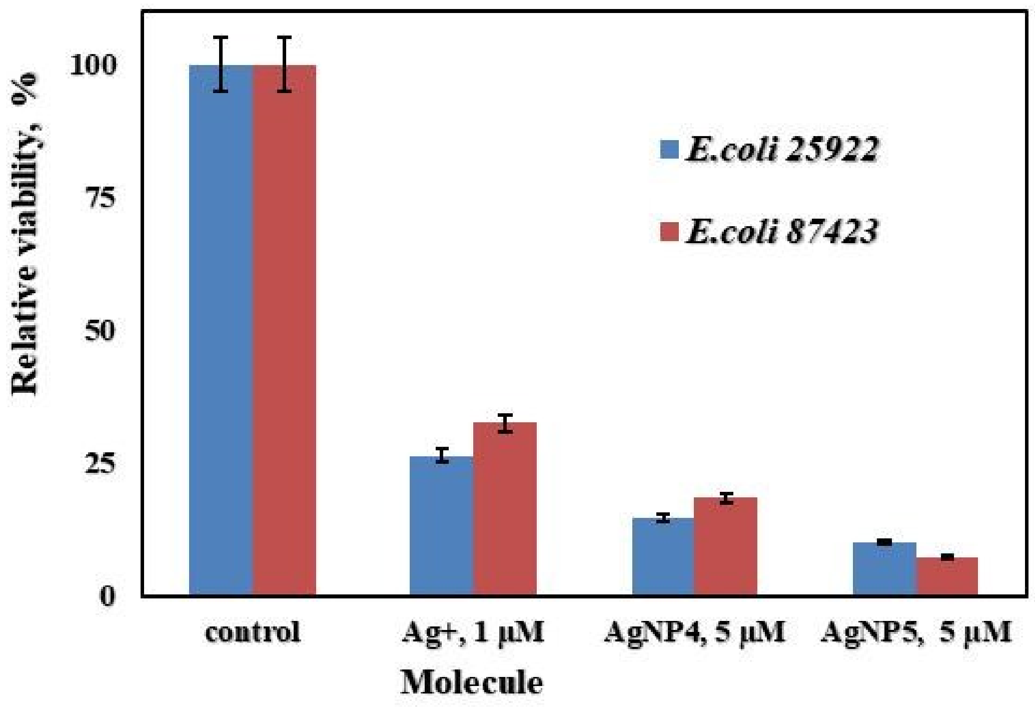

Figure S4: Silver ion was also compared with QDP-AgNPs; similar to *Trichaptum biforme*, *E.coli* 87423 and *E.coli* 25922 promoted the synthesis of silver nanoparticles from the silver ion in the media resulting in lower toxicity than expected.

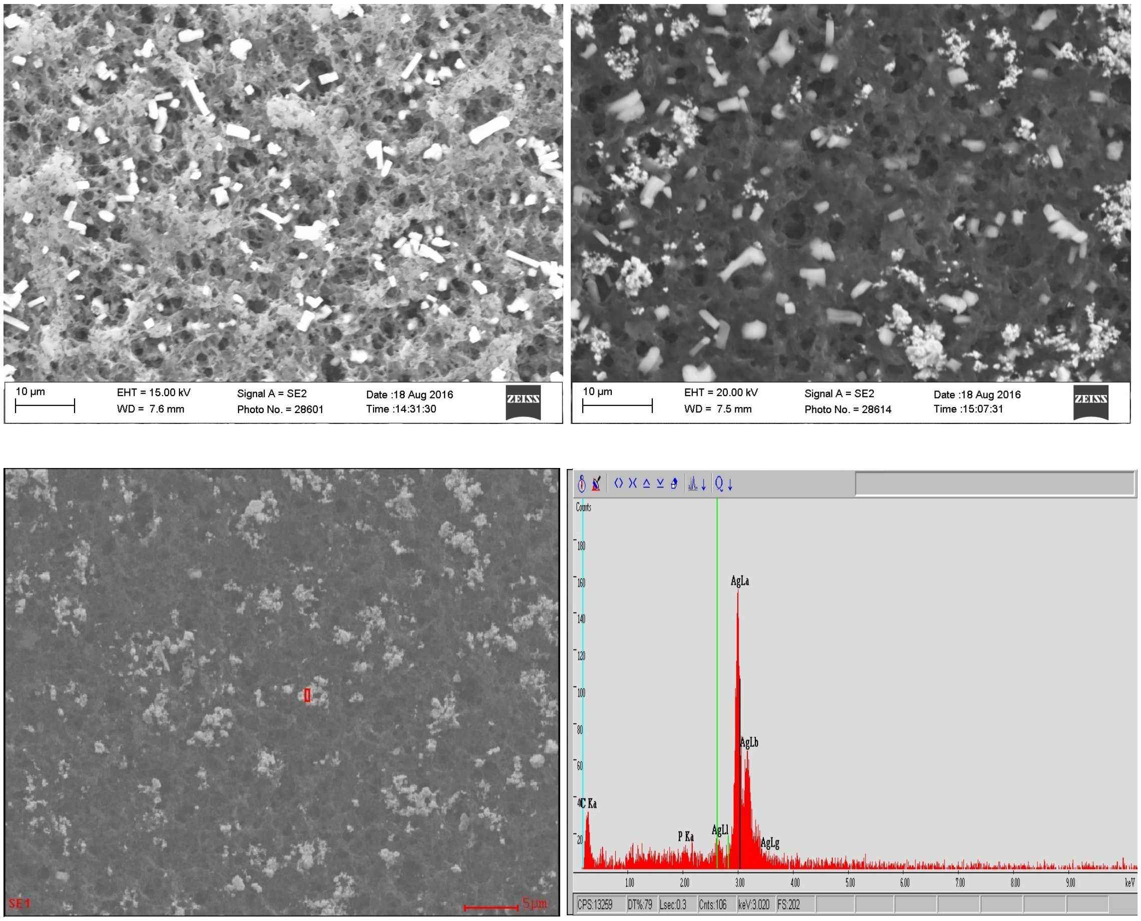

Figure S5: SEM images of *E.coli* 25922 (control) and E.coli 25922 treated with QDP-AgNP4. The shape of bacteria in both cases seems atypical which can be related to sample preparation. Localized accumulations of QDP-AgNP4 nanoparticles were observed. No change on bacterial surface was observed. EDX screening showed that individual *E.coli* 25922 was giving silver peak, which could be from silver nanoparticle or the silver ion formed from the silver nanoparticle.

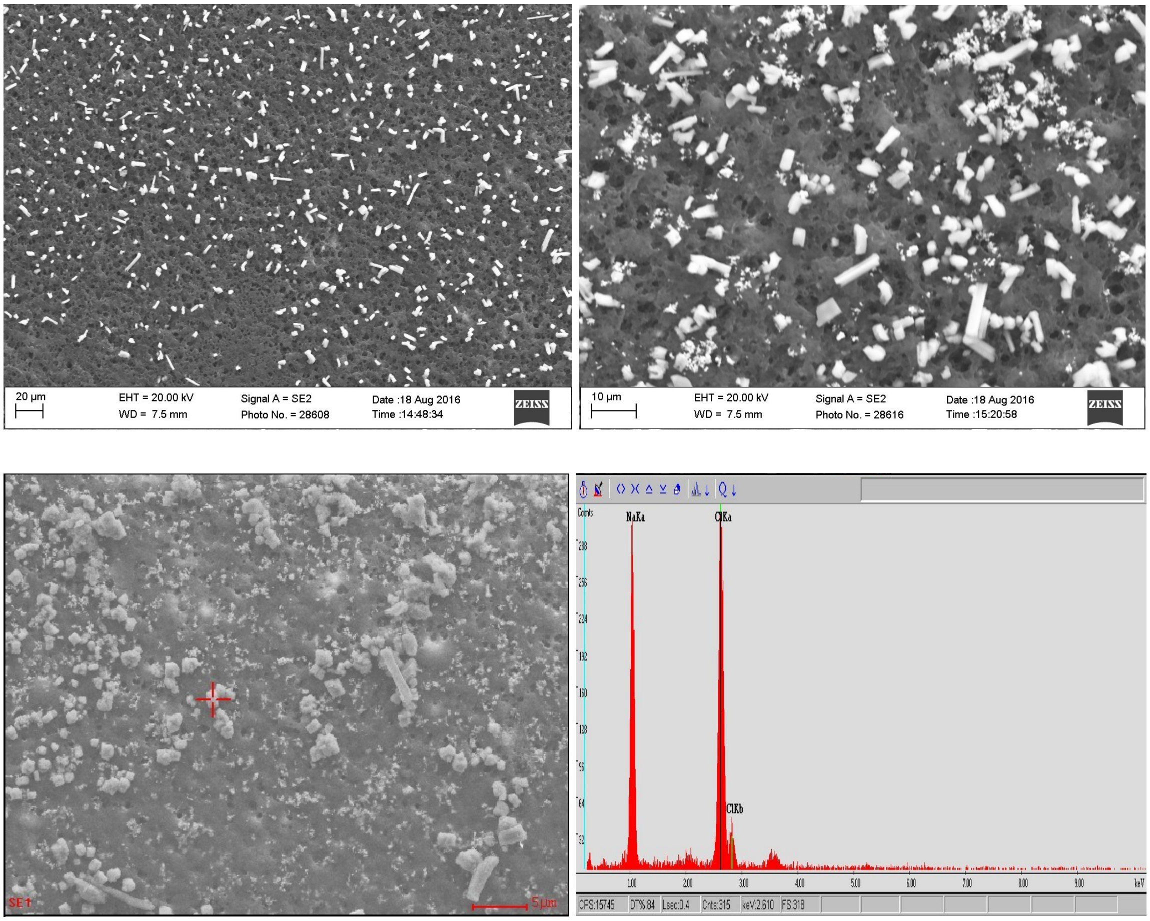

Figure S6: SEM images of *C.freundii* (control) and *C.freundii* treated with QDP-AgNP4. The shape of bacteria in both cases seems atypical, which can be related to sample preparation. Localized accumulations of QDP-AgNP4 nanoparticles were observed. Disruptions on bacterial surface were observed. EDX screening showed that individual *C.freudii* was not giving any silver peak, which could be from silver nanoparticle or the silver ion formed from the silver nanoparticle.

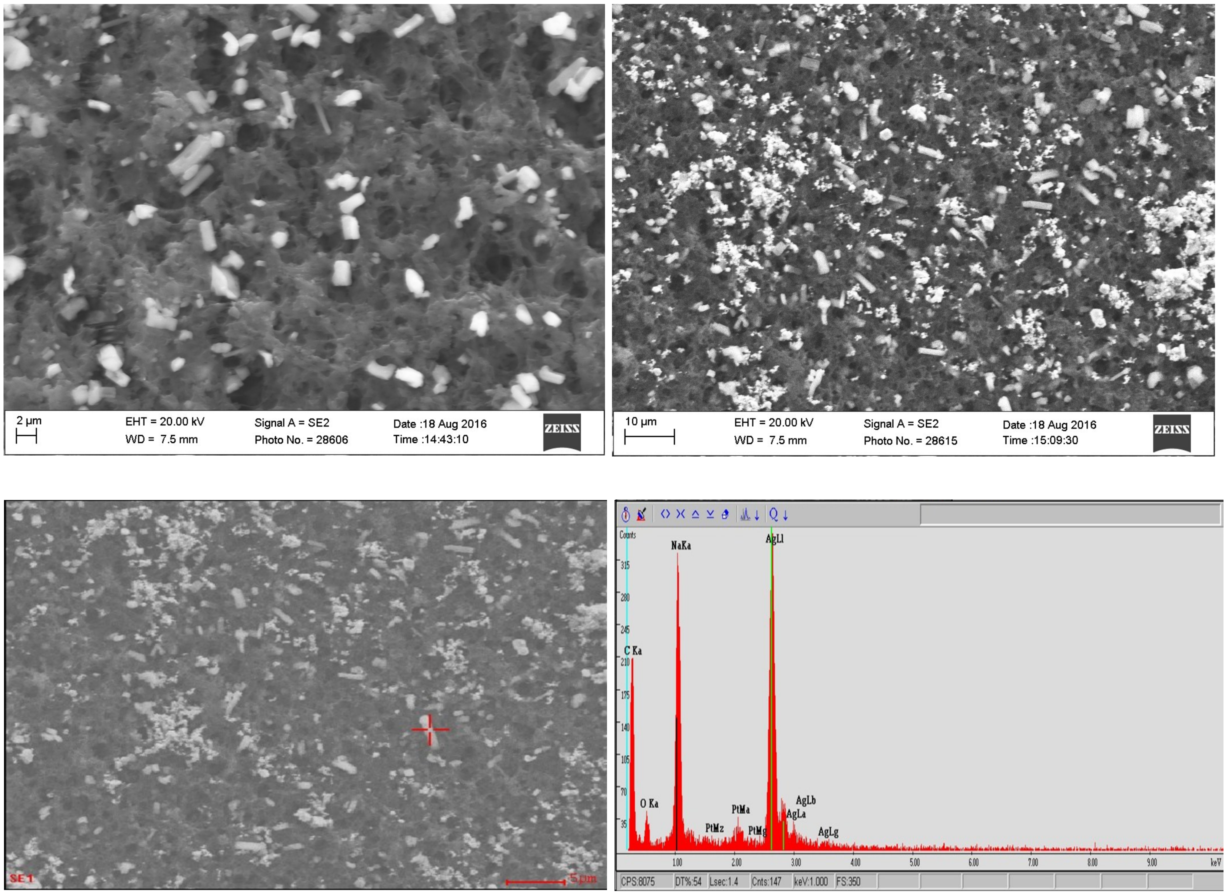

Figure S7: SEM images of *S.epidermidis* (control) and S.epidermidis treated with QDP-AgNP4. The shape of bacteria in both cases seems atypical, which can be related to sample preparation. Localized accumulations of QDP-AgNP4 nanoparticles were observed. No change on bacterial surface was observed. EDX screening showed that individual *S.epidermidis* was giving silver peak, which could be from silver nanoparticle or the silver ion formed from the silver nanoparticle.

EDX characterization was conducted for QDP-AgNP5; and the results clearly showed that no silver peak was observed for *E.coli* 25922 whereas *C.freundii* and *S.epidermidis* gave silver peak. Since it was not possible to screen all of the individual bacteria, 10 isolated cells were targeted to check the presence of silver peaks. When the same amount of QDP-AgNP4 was added to the *E.coli* solution which was then readily dried for SEM studies, no sign of silver ion/nanoparticle presence was recorded within the cells. Recently, Butler *et al* (2015) reported there must be an incubation period for nanoparticles to get into cells.[35] Overall the current results show that silver nanoparticle or silver ions did not necessarily get into bacterial cells, and the uptake of silver nanoparticles/silver ions were not dependent on gram type of the bacteria tested.

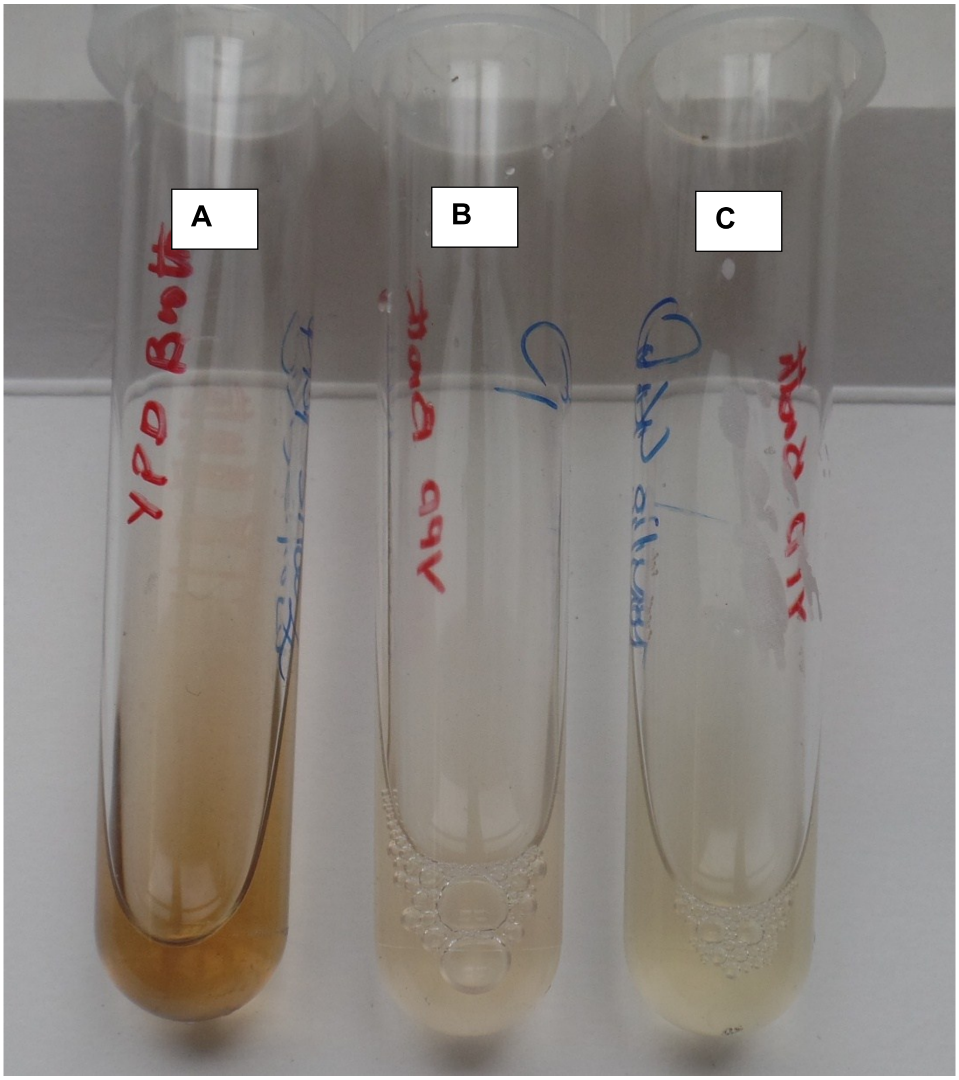

Figure S8: 10^4 cells/mL of *Trichaptum biforme* was grown in YPD-Broth. QDP-AgNP4 (A), QDP-AuNP (B) and QDP-AgNP1(C) were at 10^{-4} M concentration added to each container. Only, QDP-AgNP4 showed the elimination of *Trichaptum biforme*. When similar test was performed for *A.nidulans*, AgNP4 showed total elimination whereas in the presence of AgNP-5, up to 99% elimination was recorded.
